# Surgical outcomes of endoscopic endonasal surgery for nonfunctioning pituitary adenoma in elderly patients: a comprehensive analysis beyond age: Surgery for pituitary adenoma among elderly patients

**DOI:** 10.1186/s12902-026-02173-6

**Published:** 2026-02-12

**Authors:** Sun Mo Nam, Jong Ha Hwang, Hye Seok Park, Seung Shin Park, Jung Hee Kim, Min-Sung Kim, Chul-Kee Park, Hee-Pyoung Park, Yong Hwy Kim

**Affiliations:** 1 Department of Neurosurgery, Seoul National University Hospital, Seoul National University College of Medicine, 101 Daehak-ro. Jongno-gu, Seoul, 03080 Republic of Korea; 2 Division of Endocrinology and Metabolism, Department of Internal Medicine, Seoul National University Hospital, Seoul National University College of Medicine, Seoul, 03080 Republic of Korea; 3 Pituitary Center, Seoul National University Hospital, Seoul, 03080 Republic of Korea; 4 Department of Anesthesiology and Pain Medicine, Seoul National University Hospital, Seoul National University College of Medicine, 101 Daehak-ro, Seoul, 03080 Republic of Korea

**Keywords:** Pituitary neoplasm, Elderly patients, Endoscopic surgery, Age assessment, Surgical outcomes

## Abstract

**Background:**

Due to the rapid aging of the global population, it is challenging to identify elderly individuals who are optimal candidates for the surgical treatment of pituitary neuroendocrine tumors. While chronological age has traditionally influenced surgical decision-making, the independent effect of age on outcomes requires a comprehensive evaluation that controls for comorbidities and tumor characteristics.

**Methods:**

This retrospective analysis included 305 patients (≥18 years) who underwent endoscopic endonasal surgery for clinically nonfunctioning pituitary neuroendocrine tumors between March 2020 and February 2024 at a single tertiary center. Patients were stratified by age (<65 vs. ≥65 years) and evaluated using propensity score matching. The outcomes included gross total resection rates, complications, endocrinological recovery, and visual outcomes. Composite outcome measures were developed to assess multifaceted surgical success. Maximally selected rank statistics identified optimal age cutoffs for hormone recovery.

**Results:**

The cohort included 305 patients. Elderly patients (≥65 years, n=105) presented with significantly greater comorbidity burdens, including hypertension and diabetes. Following propensity score matching (n=105 per group), no significant age-based differences were observed in terms of gross total resection rates (84.8% vs. 84.8%) or major complications. Age 57 was found to be the optimal cutoff for endocrinological recovery (60.7% vs. 33.1%, p<0.001), with the gonadotroph axis being the most significantly affected hormone axis. The visual outcomes demonstrated remarkable age independence, as recovery was mainly dependent on gross total resection and preoperative visual field severity. Multivariate analysis demonstrated that higher body mass index (BMI), specific cardiovascular comorbidities, and intraoperative cerebrospinal fluid leaks, rather than chronological age, served as independent predictors of adverse outcomes, including surgical site infections and prolonged hospital stays.

**Conclusion:**

Chronological age alone should not constitute an absolute contraindication to endoscopic pituitary surgery. These findings underscore that comprehensive preoperative evaluations should prioritize functional status and modifiable risk factors, including cardiovascular comorbidities and BMI, to optimize outcomes for patients of all ages.

**Supplementary Information:**

The online version contains supplementary material available at 10.1186/s12902-026-02173-6.

## Introduction

There is ongoing debate in modern medicine regarding whether surgical outcomes are more strongly predicted by chronological age or functional factors. This discussion has become increasingly pertinent because the growing elderly population, the definition of “elderly” in the medical context, and the transition to superaged societies raise complex issues in terms of healthcare economics and individualized patient care. Recent discourse has emphasized comprehensive functional assessments over chronological age alone, especially in the context of surgical decision-making, thereby driving an evolution beyond simple operative outcomes to include in-depth analyses of factors that influence long-term prognosis and quality of life [1–3]. Globally, rapidly aging societies constitute an emerging problem that is no longer exclusive to high-income countries. The population aged 65 years or over will double in the next 30 years, with the global life expectancy reaching 73.3 years in 2023; however, healthy life expectancy or disability-free life expectancy has not equally increased compared with overall life expectancy [4, 5]. Consequently, the management of elderly patients has transcended mere social phenomena to become deeply embedded in clinical practice.

Pituitary neuroendocrine tumors (PitNETs), also known as pituitary adenomas, represent one of the most common intracranial neoplasms worldwide, with reported incidence rates ranging from 3.9 to 7.4 per 100,000 people across different populations; these tumors account for 10–17% of primary central nervous system tumors, and their prevalence continues to rise in parallel with population aging [6–8]. Since the widespread adoption of endoscopic endonasal surgery (EES), multiple studies have demonstrated the feasibility and safety of pituitary surgery in elderly patients [9–16]. However, few studies have comprehensively evaluated age as an independent risk factor while controlling for comorbidities and tumor characteristics. This gap is particularly significant when considering the primary surgical indications for pituitary adenomas: visual recovery and the restoration of pituitary function [10, 17]. With respect to endocrinological recovery, while postoperative functional improvement appears comparable across age groups, careful distinction must be made between age-related natural hormone decline and pathological hypopituitarism. Furthermore, the relationship between life expectancy and symptom manifestation requires careful consideration in treatment planning [11]. Although surgical outcomes, including gross total resection (GTR) rates and complications such as cerebrospinal fluid (CSF) leaks and infections, have not been reported to be significantly elevated in elderly populations, the prolonged recovery periods and functional status changes characteristic of aging warrant additional investigation [1, 3, 14, 18]. Therefore, this study aims to comprehensively analyze surgical outcomes and complication rates following EES in elderly patients and to identify preoperative independent risk factors for adverse outcomes. Through this analysis, we seek to elucidate the role of age in surgical prognosis and to establish evidence-based criteria for precise patient selection and counseling in the elderly population.

## Material and methods

### Study design and patient selection

This retrospective study included patients aged ≥18 years who underwent EES for clinically nonfunctioning PitNETs between March 2020 and February 2024 at a single tertiary center. The institutional review boards (IRBs) of Seoul National University Hospital (SNUH) approved this study (SNUH number: 2507–164-1659). All patients underwent comprehensive preoperative endocrinological, ophthalmological, rhinological, and neurological evaluations, as well as follow-up assessments, as described in our previous reports [19]. Patients with incomplete endocrinological or radiological data or those who received combined transcranial‒endonasal approaches were excluded. In total, 305 patients were eligible for the study.

### Surgical procedures

All procedures were performed using the EES technique as previously described [19, 20]. In brief, the surgical approach was individualized based on tumor extent and invasiveness. For Knosp grade 4 tumors, wide exposure included posterior ethmoidectomy and bone removal over the internal carotid artery. Tumor resection utilized extracapsular dissection if possible, with cavernous sinus exploration performed when invasion was suspected. With subarachnoid space invasion, the “second-floor approach” was utilized, thus enabling direct tumor dissection from above through an intentional incision in the diaphragma sellae [20]. Reconstruction was performed using multilayer techniques with fibrin sealants for low-flow CSF leaks and pedicled nasoseptal flaps for high-flow leaks [21].

### Clinical criteria

Visual outcomes were evaluated using established methodologies from previous studies. Visual impairment and visual field deficits, including the Visual Impairment Score (VIS) and the Visual Field Score (VFS), were assessed via the German Ophthalmological Society scoring system [22]. The visual fields were evaluated using kinetic Goldmann perimetry and/or a Humphrey field analyzer. Preoperative and postoperative endocrinological status was analyzed by a multidisciplinary pituitary team, including endocrinologists [19, 23]. Preoperative hormone replacement therapy and dopamine agonist use were systematically documented. The diagnostic protocol followed the Endocrine Society guidelines [24], applying Korean-specific reference ranges for insulin-like growth factor-1 [25].

Tumor volume was estimated using the modified ellipsoid formula (A×B×C/2) based on the maximal 3-dimensional diameter, and tumors with a maximal diameter ≥4 cm were defined as giant PitNETs.

Chronic kidney disease (CKD) was defined as an estimated glomerular filtration rate (eGFR) lower than 60 mL/min/1.73 m^2^. Liver cirrhosis (LC) included all Child‒Pugh classes A, B, and C; however, our surgical cohort included only Child‒Pugh class A patients. Heart disease was categorized as coronary artery disease (CAD), valvular disease, or arrhythmia. The American Society of Anesthesiologists (ASA) physical status classification was evaluated by anesthesiologists following routine practice before surgery. Diabetes insipidus (DI) was categorized as transient or persistent DI based on the 3-month post-operative evaluation. Hyponatremia was defined as a serum sodium concentration lower than 135 mEq/L within 3 months. Detailed diagnostic criteria and management protocols followed as described in our previous report [26]. Delirium was considered when it required medical intervention. Hospital length of stay (LOS) was categorized as prolonged when it was ≥5 days because our institutional median length of stay was 4 days [12, 27]. Additionally, composite outcomes were defined to assess overall surgical success based on the international core outcome set for pituitary surgery research [28]. A “good outcome” was defined as meeting all of the following criteria: the absence of surgical site infection, reoperation, or readmission; an LOS of less than 5 days; no worsening of visual function; and no new postoperative hormone deficits. Conversely, a “suboptimal outcome” was defined as the presence of any postoperative complication (including but not limited to DI, hyponatremia, surgical site infection, or delirium), any worsening of vision, any new hormone deficit, or failure to achieve GTR. The rates of these composite outcomes were compared between age groups using the chi-square test.

To account for surgical complexity, operative approaches were categorized based on anatomical corridors [29]. Standard trans-sellar approaches were classified as basic procedures, whereas expanded endonasal approaches extending beyond the sella were categorized by their primary corridors: sagittal extensions (trans-tuberculum, trans-planum, trans-clival) and coronal extensions (trans-pterygoid, trans-sphenopalatine). Cases utilizing multiple corridors were classified via combined approaches.

### Statistical analysis

All the statistical analyses were performed using R (Version 4.5.3, R Foundation for Statistical Computing, Vienna, Austria). This was a consecutive case series; no formal sample size calculation was performed. The patient cohort was stratified by age ( < 65 years vs. ≥65 years). Baseline characteristics were compared using the Mann‒Whitney U test for continuous variables and the chi‒square test or Fisher’s exact test for categorical variables. A two-sided *p* value < 0.05 was considered statistically significant.

To evaluate the independent effect of age, propensity score matching (PSM) analysis was performed using optimal matching (1:1 ratio) based on preoperative covariates, including demographics (sex, body mass index [BMI]), functional status (ASA classification), comorbidities (diabetes mellitus [DM], hypertension [HTN], dyslipidemia [DL], CKD, chronic obstructive pulmonary disease [COPD], LC, smoking status, neuropsychiatric medication use, history of malignancy, and heart disease), and tumor characteristics (maximal diameter, Knosp grade, and recurrence status). Covariate balance was assessed using standardized mean differences (Additional file 1). For outcomes that showed a suggestive association (*p* < 0.2) after PSM, multivariable regression analyses were subsequently performed on the entire cohort to identify independent risk factors and maximize statistical power. A two-step variable selection process was employed, with initial univariable screening (*p* < 0.2) followed by final multivariable modeling.

Model selection was tailored to each outcome variable’s distribution: negative binomial regression for LOS to account for overdispersion; Firth’s penalized logistic regression for the rare event of surgical site infection (SSI); standard logistic regression for other binary outcomes, including hyponatremia and GTR achievement; and proportional odds ordinal logistic regression for ordinal visual outcomes. An optimal age cutoff point for composite recovery outcomes was estimated using maximally selected rank statistics (maxstat) testing, with bootstrap validation (1,000 replications) to assess threshold stability. For hormone axes demonstrating age effects, multivariable logistic regression models were constructed following the same covariate selection process described above. Missing data were handled by complete case analysis.

## Results

### Patients and tumor characteristics

Among the 305 patients, the median age was 57 years (range 21–84), and 166 (54.4%) were male (*p* = 0.544). Elderly patients (≥65 years, *n* = 105) demonstrated significantly greater comorbidity burdens, including DM (28.6% vs. 9.5%, *p* < 0.001), HTN (54.3% vs. 27.0%, *p* < 0.001), and CAD (12.4% vs. 2.0%, *p* < 0.001). However, there were no significant differences in tumor size, volume, or Knosp grade invasiveness (Table 1). At baseline, patients with established pituitary deficiencies were receiving appropriate hormone replacement therapy in accordance with institutional protocols. Sex hormone replacement was prescribed in a minority of selected cases. Additionally, four patients were prescribed dopaminergic agonists at other hospitals for suspected prolactinomas but were confirmed to have nonfunctioning adenomas with stalk effect upon re-evaluation at our center. One patient underwent gamma knife radiosurgery before tissue confirmation. The detailed comorbidity distributions by 10-year age groups are provided in Additional file 2 and 3.

**Table 1:** Demographic and clinical characteristics of study participants stratified by age group (*n* = 305)

	Overall	Aged < 65 yrs	Aged ≥ 65 yrs	p^1^
	305	200	105	
**Demographics**				
Age	57 [47,67]	50 [42, 57]	71 [67, 74]	
Sex (Male:Female)	166:139 (54.4:45.6:)	107:93 (53.5:46.5)	59:46 (56.2:43.8)	0.743
BMI	25.0 [23.0, 28.0]	25.5 [23.0, 28.0]	25.0 [24.0, 27.0]	0.522
**Comorbidities**				
DM	49 (16.1)	19 (9.5)	30 (28.6)	< 0.001
HTN	111 (36.4)	54 (27.0)	57 (54.3)	< 0.001
DL	109 (35.7)	61 (30.5)	48 (45.7)	**0.012**
CKD	12 (3.9)	2 (1.0)	10 (9.5)	**0.001**^**2**^
COPD	14 (4.6)	4 (2.0)	10 (9.5)	**0.007**^**2**^
LC	2 (0.7)	0 (0.0)	2 (1.9)	**0.118**^**2**^
Heart				< 0.001
CAD	17 (5.6)	4 (2.0)	13 (12.4)	
Valve	1 (0.3)	1 (0.5)	0 (0.0)	
Arrythmia	6 (2.0)	1 (0.5)	5 (4.8)	
Smoking				0.077
Current	42 (13.8)	34 (17.0)	8 (7.6)	
Ex-smoker	17 (5.6)	11 (5.5)	6 (5.7)	
NP	13 (4.3)	6 (3.0)	7 (6.7)	0.145^2^
Malignancy Hx.	35 (11.5)	18 (9.0)	17 (16.2)	0.092
ASA				**0.002**^**2**^
Class 1	24 (7.9)	22 (11.0)	2 (1.9)	
Class 2	270 (88.5)	174 (87.0)	96 (91.4)	
Class 3	11 (3.6)	4 (2.0)	7 (6.7)	
**Preoperative Functional Status**				
**Preoperative Visual Impairment**	219 (72.3)	138 (69.7)	81 (77.1)	0.214
**VIS**^**2**^	6.0 [2, 20]	4.0 [0, 20]	10.0 [4, 20]	**0.001**
**VFS**^**2**^	5.0 [0, 18]	5.0 [0, 18]	10.0 [4, 18]	0.122
**Preoperative Pituitary Axis Abnormalities**				
Gonadotroph	223 (73.1)	137 (68.5)	86 (81.9)	**0.018**
Somatotroph	206 (67.5)	123 (61.5)	83 (79.0)	**0.003**
Corticotroph	72 (23.6)	40 (20.0)	32 (30.5)	0.057
Thyrotroph	31 (10.2)	19 (9.5)	12 (11.4)	0.741
Lactotroph	61 (20.0)	47 (23.5)	14 (13.3)	0.050
**Tumor characteristics**				
Size				0.110
Micro	4 (1.3)	4 (2.0)	0 (0.0)	
Macro	269 (88.2)	179 (89.5)	90 (85.7)	
Giant	32 (10.5)	17 (8.5)	15 (14.3)	
Volume	5.83[3.85, 9.36]	6.04 [3.84, 9.34]	5.51 [3.85, 9.36]	0.715
Knosp Grade				0.427
Grade 0	24 (7.9)	20 (10.0)	4 (3.8)	
Grade 1	85 (27.9)	53 (26.5)	32 (30.5)	
Grade 2	78 (25.6)	50 (25.0)	28 (26.7)	
Grade 3	73 (23.9)	48 (24.0)	25 (23.8)	
Grade 4	45 (14.8)	29 (14.5)	16 (15.2)	
Recurred tumor	43 (14.1)	32 (16.0)	11 (10.5)	0.253
Previous Treatment				0.186
OP only	40 (13.1)	30 (15.0)	10 (9.5)	
OP + GKRS	1 (0.3)	1 (0.5)	0 (0.0)	
Op + RT	1 (0.3)	1 (0.5)	0 (0.0)	
Op + RT + GKRS	1 (0.3)	1 (0.5)	0 (0.0)	

The data are presented as the n (%) or median (interquartile range [IQR])

^1^ *p* values were calculated using the chi-square test or Fisher’s exact test for categorical variables and the Mann‒Whitney U test for continuous variables

^2^ Quantitative visual assessments were analyzed in patients with available data (Visual Impairment Score: *n* = 272; Visual Field Score: *n* = 303)

*Abbreviations:* ASA, American Society of Anesthesiologists; BMI, body mass index; CAD, coronary artery disease; CKD, chronic kidney disease; COPD, chronic obstructive pulmonary disease; DL, dyslipidemia; DM, diabetes mellitus; GKRS, Gamma Knife radiosurgery; HTN, hypertension; Hx, history; IQR, interquartile range; LC, liver cirrhosis; NP, neuropsychiatric medication; OP, operation; RT, radiotherapy; VFS, Visual Field Score; VIS, Visual Impairment Score

Tumor pathology differed significantly between age groups (*p* = 0.011), with gonadotroph tumors (steroidogenic factor 1 lineage) accounting for 62.9% of cases among elderly patients and 44.5% among younger patients. Conversely, PIT1-lineage tumors were less common in elderly patients (2.9% vs. 9.5%). Furthermore, the Ki-67 proliferation index was significantly lower in elderly patients (median 1.80 vs. 2.40, *p* < 0.001) (Additional file 4 and 5).

### Surgical outcomes

Overall GTR was achieved in 259 patients (84.9%). Six patients (2.0%) underwent reoperation, and 13 patients (4.3%) were readmitted. The most common complication was DI (*n* = 96, 31.5%), which presented as transient DI in 75 patients (24.6%) and persistent DI in 21 patients (6.9%). Hyponatremia was observed in 81 patients (26.6%), and surgical site infection occurred in 9 patients (3.0%). Deep vein thrombosis/pulmonary embolism and mortality were not observed in this cohort (Additional file 6).

**Fig. 1:**
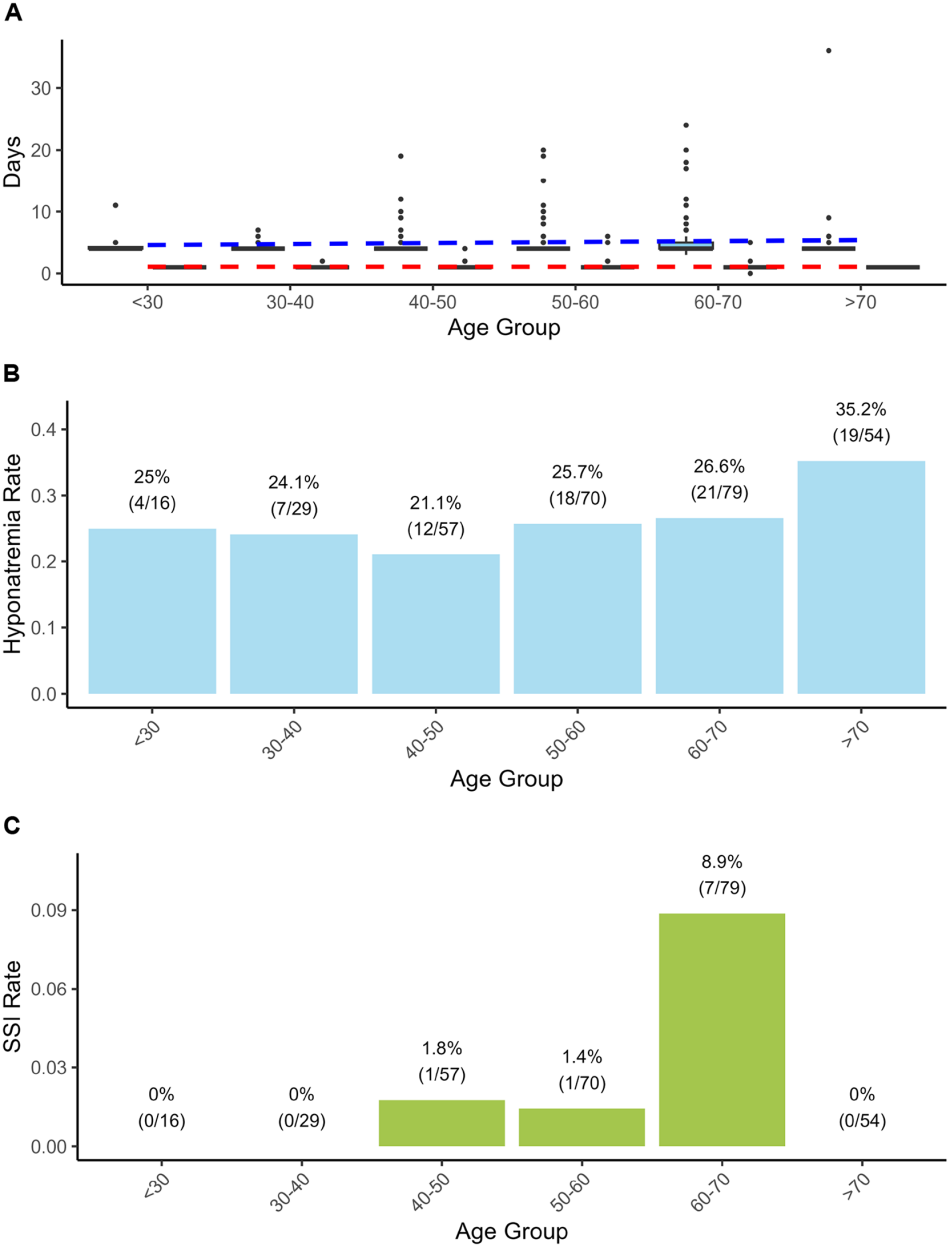
Age-related surgical outcomes following endoscopic skull base surgery. (**A**) Hospital length of stay (Los,blue boxplots) and intensive care unit length of stay (ICU, red boxplots) by age group. Box plots show median values (solid lines), interquartile ranges (boxes), and outliers (individual dots). While median stays remained consistent across all age groups (hospital stay: 4 days; ICU stay: 1 day), elderly patients ( > 70 years) demonstrated greater variability with more frequent prolonged hospitalizations. (**B**) Hyponatremia rates across age groups, showing relatively stable incidence ranging from 21.1% to 35.2%. The highest rate was observed in patients over 70 years of age (35.2%, 19/54 patients). Sample sizes for each age group are indicated in parentheses. (**C**) Surgical site infection (SSI) rates by 10-year age groups. Peak incidence occurred in the 60–70 years age group (8.9%, 7/79 patients), with no infections observed in patients under 40 years or over 70 years of age. Abbreviations: HD, hospital days; ICU, intensive care unit; SSI, surgical site infection

PSM analysis revealed that GTR rates and the total number of complications did not significantly differ by age (Table 2). However, surgical site infection (*p* = 0.12), prolonged LOS (*p* = 0.20), and hyponatremia (*p* = 0.12) tended to be more common in elderly individuals (Fig. 1). The tailored regression models revealed that age itself was not a significant predictor of any surgical outcome, including the GTR rate, incidence of surgical complications or infection, or LOS. (Detailed Results in Additional file 7). DL exerted a significant protective effect against overall complications (OR 0.58, 95% CI 0.34–0.97, *p* = 0.047), with all DL patients receiving preoperative statin therapy. However, no other factors exhibited significant associations with surgical complications. For hyponatremia prediction, the logistic regression model (AUC 0.71, Pseudo R^2^ 0.091) identified transient DI (*p* = 0.030) and persistent DI (*p* = 0.023) as significant risk factors. Notably, a higher BMI was found to have a significant protective effect against hyponatremia (*p* = 0.048). The LOS was influenced by higher BMI, COPD, recurrent tumor status, complications, surgical complexity, cavernous sinus exploration, and intraoperative CSF leakage (AIC 1309.9, Pseudo R^2^ 0.396). Risk factors for surgical site infection included coronary artery disease, arrhythmia, higher BMI, and intraoperative CSF leakage (AUC 0.914, McFadden’s pseudo R^2^ 0.05).

**Table 2:** Surgical outcomes after propensity score matching by age group (*n* = 105 per group)

Variable	Aged < 65 yrs	Aged ≥ 65 yrs	OR (95% CI)	*p*
GTR rate	89 (84.8)	89 (84.8)	1.00 (0.44–2.28)	1.000
Major Complications	6 (5.7%	10 (9.5)	1.73(0.55–6.04)	0.436
SSI	1 (1.0)	6 (5.7)	6.26 (0.74–292.25)	0.119
Readmission	4 (3.8)	5 (4.8)	1.26 (0.26–6.55)	1.000
Reoperation	2 (1.9)	1 (1.0)	0.50 (0.01–9.68)	1.000
Minor Complications	60 (57.1)	68 (64.8)	1.38 (0.76–2.50)	0.322
DI	35 (33.3)	37 (35.2)	1.09 (0.59–2.00)	0.885
Hyponatremia	23 (21.9)	34 (32.4)	1.70 (0.88–3.33)	0.12
Delirium	4 (3.8)	7 (6.7)	1.80 (0.44–8.65)	0.538
Pneumonia	0	1 (1.0)	-	-
LOS	4 [4, 4]	4 [4, 5]	0.73 (0.24–1.7)	0.138
ICU	1 [1, 1]	1 [1, 1]	0.01 (0.00–0.14)	0.898
Prolonged LOS	22 (21.0)	31 (29.5)	1.58 (0.8–3.13)	0.204

Data are presented as n (%) for categorical variables and median (interquartile range [IQR]) for continuous variables

Propensity score matching (1:1 ratio) was performed using preoperative covariates to balance baseline characteristics between age groups

P-values were calculated using Fisher’s exact test for categorical variables and Welch’s t-test for continuous variables. Odds ratios were estimated using logistic regression

*Abbreviations*: CI, confidence interval; DI, diabetes insipidus; GTR, gross total resection; ICU, intensive care unit; IQR, interquartile range; LOS, length of stay; OR, odds ratio; SSI, surgical site infection

### Endocrinological and visual outcomes

**Fig. 2:**
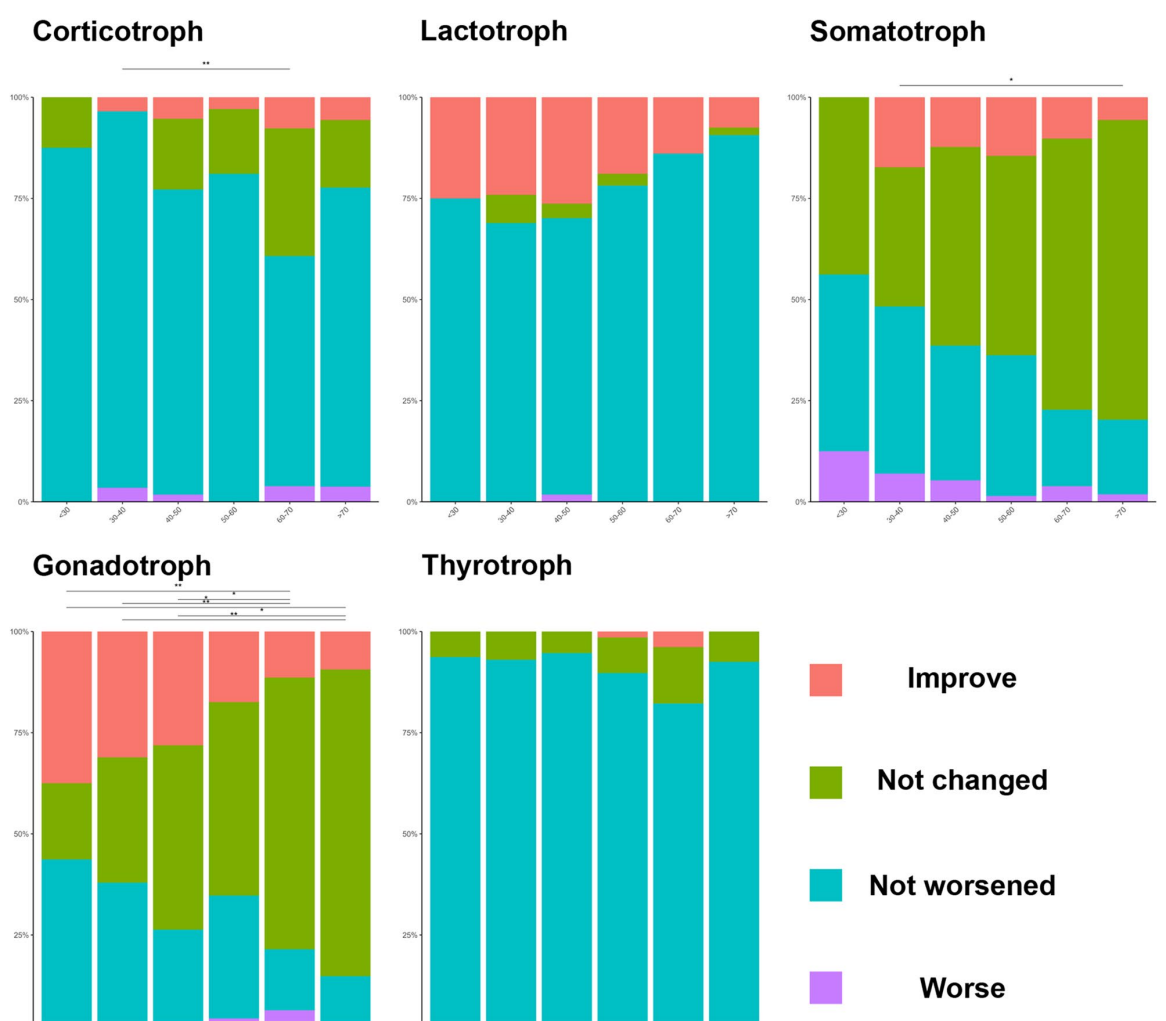
Age-related endocrinological recovery patterns by individual hormone axis Bar charts showing the distributions of postoperative hormone outcomes across age groups (10-year intervals) for five pituitary hormone axes. Each bar represents 100% of patients with preoperative hormone deficiency in that age group. The colors indicate the following: improvement (red) - recovery from preoperative deficiency to normal function; no change (green) - persistent deficiency without deterioration; no worsening (cyan) - stable function in patients with borderline or partial deficiency; and worsening (purple) - new onset deficiency or deterioration of existing deficiency. Statistical significance was determined by Fisher’s exact test for age group comparisons: *p < 0.05, **p < 0.01, ***p < 0.001

Endocrinological outcomes stratified by 10-year age groups revealed significant age-related differences in hormone recovery patterns (Fig. 2, detailed pairwise comparisons in Additional file 8). Patients over 70 years of age showed markedly poor recovery: gonadotroph axis improvement declined from 37.5% ( < 30 years) to 9.3% (*p* = 0.002), and somatotroph axis recovery decreased from 17.2% to 5.6% (*p* = 0.039). In contrast, corticotroph axis recovery was marginally significantly greater in the 60- to 70-year-old group than in the 30- to 40-year-old group (*p* = 0.005) but was consistently poor across all ages (0–7.6%). The thyrotroph and lactotroph axes showed no statistically significant differences in recovery rates based on age after correction for multiple comparisons. Among all hormone axes, the gonadotroph axis consistently presented the highest recovery potential across all ages, whereas the thyrotroph axis presented minimal recovery regardless of age (0–3.8%).

To identify optimal age thresholds for hormone recovery, we performed maximally selected rank statistics analysis with validation. Age 57 was identified as the most significant cutoff for hormone recovery (*p* < 0.001), with patients under 57 years showing a recovery rate of 60.7% compared with 33.1% in those aged 57 and above. Bootstrap validation (1,000 replications) and multivariable regression analysis for individual hormone axes confirmed age-dependent patterns (Additional file 9). Bootstrap analysis demonstrated stable thresholds: overall hormone recovery showed a mean cutoff of 60.1 years (95% CI 47–70), gonadotroph axis 46.5 years (95% CI 37.0–60.0), and somatotroph axis 56.3 years (95% CI 42.0–69.0). In multivariable logistic regression models adjusted for sex, BMI, and pathology, chronological age remained an independent predictor of recovery for both gonadotroph (aOR 0.93, 95% CI 0.90–0.96; *p* < 0.001) and somatotroph axes (aOR 0.96, 95% CI 0.93–0.99; *p* = 0.019), confirming that age-related decline is intrinsic to aging rather than secondary to tumor characteristics or surgical factors (detailed in Additional file 10).

**Fig. 3:**
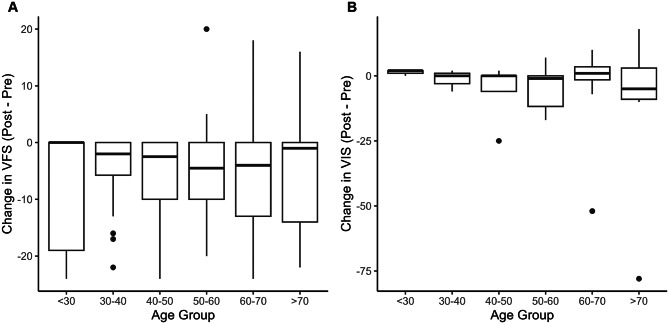
Visual outcomes following endoscopic surgery stratified by age group. (**A**) visual field Score (VFS) changes (postoperative minus preoperative scores). (**B**) Visual impairment Score (VIS) changes (postoperative minus preoperative scores). Box plots showing changes in visual function scores across age groups (10-year intervals). Visual function was assessed using the German ophthalmological Society visual scoring system. Negative values indicate improvement in visual function

Visual outcomes assessed by the VIS (*n* = 36) and VFS (*n* = 289) revealed no significant age-related differences (Fig. 3). An analysis of patients with preoperative visual symptoms and available data (*n* = 212) using ordered logistic regression revealed that a more severe preoperative VFS showed better recovery potential (OR 0.93, 95% CI: 0.88–0.98, *p* < 0.01), as did GTR (OR 0.28, 95% CI: 0.11–0.75, *p* < 0.01), whereas DM had a marginal effect (OR 2.38, 95% CI: 0.95–5.79, *p* = 0.060).

### Composite outcomes

An analysis of the designed good outcome and suboptimal outcome models revealed no significant differences between older and younger patients (*p* = 0.36 and *p* = 0.46, respectively). Maxstat analysis confirmed that there was no significant age cutoff for overall surgical or functional outcomes (*p* = 0.34 and *p* = 0.93, respectively).

## Discussion

This comprehensive analysis of 305 patients who underwent EES for nonfunctioning PitNETs revealed that chronological age alone is not a primary risk factor for neurological and surgical outcomes. However, age plays a key role in endocrinological recovery. While elderly patients presented with significantly greater comorbidity burdens, these findings challenge the traditional age-based contraindications of surgery and support a more nuanced approach to patient selection.

### Surgical outcomes and age independence

The GTR rates (84.9%) were comparable across age groups, which is consistent with the established literature showing that tumor characteristics, particularly the degree of cavernous sinus invasion and recurrent tumors, rather than patient age, determine resectability [11, 12]. The absence of mortality in this series, combined with equivalent complication rates between age groups, reinforces the safety profile of modern surgical techniques in carefully selected elderly patients [9, 11]. These findings align with recent reports demonstrating the feasibility of pituitary surgery when performed at high-volume centers [12, 27].

Multivariate analysis revealed that patient-specific comorbidities, rather than chronological age, are risk factors for surgical complications. This finding supports the growing emphasis on functional status and frailty indices over chronological age in surgical decision-making, which have demonstrated superior predictive value compared with traditional scoring systems in other surgical subspecialties [3, 30, 31]. While formal frailty assessment tools (e.g., grip strength, gait speed, modified frailty index) were not available in our retrospective cohort, we utilized ASA classification and comprehensive comorbidity profiling as surrogate markers of physiological reserve. Our approach of incorporating multiple comorbidity domains (cardiovascular, metabolic, pulmonary, renal) along with functional status (ASA) provides a multidimensional assessment that approximates frailty evaluation.

### Complication patterns and risk factors

The protective effect of DL likely represents confounding by indication, as DL patients receiving statin therapy have better outcomes because of the pleiotropic benefits of the medication rather than the underlying disorder itself. This phenomenon has been well documented in various surgical populations [32, 33].

Surgical site infection analysis revealed potential insights into risk factor patterns. While intraoperative CSF leakage remains the most established predictor [21, 27, 34], our findings that coronary artery disease and arrhythmias independently predict SSI suggest possible underlying mechanisms that warrant further investigation. Heart disease might serve as a surrogate marker for systemic inflammatory status and compromised wound healing capacity, which is particularly relevant in the sinonasal environment where mucosal integrity is crucial for infection prevention [35–37]. The established association between chronic rhinosinusitis and cardiovascular disease provides indirect support for this hypothesis; however, prospective studies are critically needed to validate these relationships [38]. Hyponatremia patterns demonstrated complex interactions between DI, BMI, and electrolyte homeostasis. The protective effect of higher BMI against hyponatremia conflicts with some reports but is consistent with others showing BMI-dependent risk patterns for early versus delayed hyponatremia [39, 40]. These findings suggest that sodium management protocols should be individualized based on patient phenotype rather than age alone. The LOS is strongly linked to the burden of the surgical event itself—encompassing procedural complexity and postoperative complications—rather than the patient’s age [3, 18, 41]. However, while median stays remained consistent across all ages, elderly patients demonstrated greater variability, with more frequent outliers experiencing exceptionally prolonged hospitalizations. This pattern suggests that although most elderly patients recover comparably to younger patients, a subset may experience significantly complicated courses, highlighting the need for individualized risk assessment and enhanced monitoring protocols in this population [1, 3, 14, 18].

### Endocrinological and visual recovery

The identification of age 57 as a significant cutoff for endocrinological recovery represents a clinically relevant finding that merits careful interpretation. The gonadotroph and somatotroph axes demonstrated a significant age-related decline in recovery potential between specific age groups, the effect was most pronounced in the gonadotroph axis. This threshold aligns with the natural decline in gonadotroph function associated with menopause and andropause, suggesting that surgical intervention may have limited benefit for hypothalamic‒pituitary‒gonadal axis recovery in patients approaching or beyond these physiological transitions [42–45]. Similarly, a previous report revealed that patients aged ≤62.2 years with adequate preoperative growth hormone reserves were more likely to recover from the adult somatotroph axis, thus establishing age as a key predictor [46]. Age-independent recovery in lactotrophs and thyrotrophs and corticotroph axes was thought to result from mass effect relief, although the recovery potential was low in thyrothrophs and corticotrophs [44]. The differential recovery patterns between hormonal axes highlight the complexity of pituitary function restoration and suggest that surgical outcomes should be considered in the context of age-specific expectations [12, 14, 44]. This finding also suggests that while surgical intervention can be beneficial, the potential for recovery in these specific axes diminishes with age [11, 47].

Visual outcome analysis demonstrated remarkable age-independence, thereby challenging assumptions about neuroplasticity limitations in elderly patients. The preserved capacity for visual recovery, contingent primarily on GTR and preoperative visual field severity, supports aggressive surgical intervention for visual symptoms regardless of patient age [10]. This finding has particular importance given the established associations between visual impairment and cognitive decline in elderly populations [48]. However, comprehensive preoperative visual assessment, including an evaluation of concurrent age-related conditions such as cataracts, glaucoma, and macular degeneration, remains essential to optimize surgical planning and outcome prediction in elderly patients [10, 47]. Visual restoration should be prioritized as the primary therapeutic goal in elderly patients, whereas surgical decompression provides mass effect relief, benefiting both endocrinological and visual outcomes [10, 14, 44, 46, 47].

### Clinical implications and assessment tool development

Our findings demonstrate that patient comorbidities and functional status, rather than chronological age per se, serve as the primary determinants of surgical outcomes. The current ASA classification, while useful, may inadequately capture the multidimensional nature of surgical risk in elderly patients. Our composite outcome analysis, which evaluated multifaceted surgical success, including the absence of complications, expected LOS, visual preservation, and hormonal stability, demonstrated no significant age-related differences (*p* = 0.36 for good outcomes, *p* = 0.46 for suboptimal outcomes). While this represents a promising approach to comprehensive outcome assessment, clinical validation and refinement of such composite tools are necessary to establish standardized evaluation frameworks. Future studies incorporating validated frailty indices [1, 31], comprehensive geriatric assessments [2, 3], and multidimensional surgical outcome evaluations are needed to optimize patient selection and perioperative management for elderly patients undergoing endoscopic skull base surgery.

### Study limitations

This single-center retrospective analysis has inherent limitations, including potential selection bias and limited long-term follow-up. First, selection bias may exist as healthier elderly patients were likely preferentially selected for surgery, limiting generalizability to all elderly NFPA patients. While we employed PSM to balance baseline characteristics, residual confounding regarding the severity or duration of age-related comorbidities may persist. Second, the absence of standardized frailty assessment tools and patient-reported quality-of-life measures limits comprehensive outcome evaluation. Future prospective studies incorporating validated frailty indices would further refine risk stratification in elderly pituitary surgery patients. Third, subgroup analysis of the oldest-old patients (≥75 years, *n* = 21; ≥80 years, *n* = 5) was not performed due to insufficient sample size. The progressively smaller and more selected cohorts at higher age cutoffs limit adequately powered analysis. Additionally, the relatively short follow-up period may not capture delayed complications or long-term functional outcomes that could differ between age groups.

## Conclusion

In this study, chronological age should not serve as an absolute contraindication to endoscopic pituitary surgery. While elderly patients require careful comorbidity assessment and individualized surgical planning, the potential for excellent visual recovery and acceptable surgical risk profiles support surgical intervention when clinically indicated. The development of age-appropriate, function-based assessment tools will be crucial for optimizing patient selection as the global population continues to age.

## Electronic supplementary material

Below is the link to the electronic supplementary material.

Additional file 1: (Figure) Propensity score matching covariate balance: love plot with standardized mean differences.

Additional file 2: (Figure) Comorbidity distribution by age group: heat map visualization.

Additional file 3: (Table) Comorbidities by 10-year age groups.

Additional file 4: (Figure) Tumor pathology distribution by lineage and age group: pie charts and stacked bar graphs.

Additional file 5: (Table) Detailed tumor pathology distribution and Ki-67 proliferation index by age group.

Additional file 6: (Table) Surgical outcomes and complications by 10-year age groups.

Additional file 7: (Table) Multivariable regression models for postoperative outcomes.

Additional file 8: (Table) Pairwise comparisons of endocrinological status changes by 10-year age groups.

Additional file 9: (Table) Age thresholds for hormone recovery by axis: maximally selected rank statistics and bootstrap validation.

Additional file 10: (Table) Predictors of hormone recovery for each pituitary axis: multivariable logistic regression models.

## Data Availability

The datasets generated during the current study are available from the corresponding author on reasonable request.
