# Supplementary material for: Surgical outcomes of endoscopic endonasal surgery for nonfunctioning pituitary adenoma in elderly patients: a comprehensive analysis beyond age: Surgery for pituitary adenoma among elderly patients
Source: BMC Endocr Disord. 2026 Feb 12;26:69. doi: 10.1186/s12902-026-02173-6 (PMC12922220; doi:10.1186/s12902-026-02173-6)
Supplement: Supplementary file 1 — Additional file 1: (Figure) Propensity score matching covariate balance: love plot with standardized mean differences. [file 12902_2026_2173_MOESM1_ESM.pdf]

Additional file 1. Propensity score matching covariate balance: love plot with standardized mean differences.

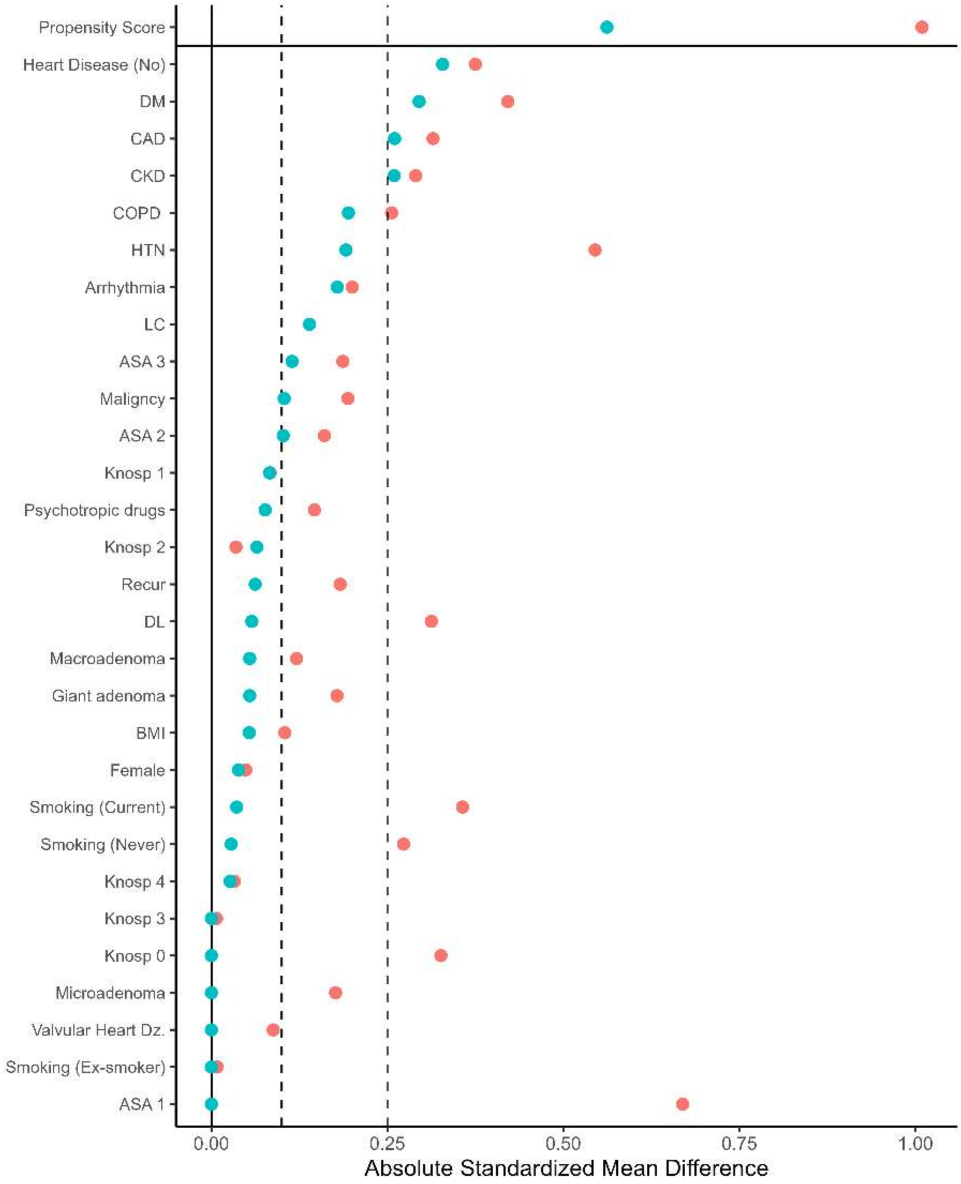

This love plot displays absolute standardized mean differences (SMD) for all covariates used in propensity score matching analysis. Dashed vertical lines indicate commonly accepted balance thresholds (SMD = 0.1 and 0.25). Red circles represent the unmatched cohort; blue triangles represent the matched cohort.

Covariates included in matching: sex, body mass index (BMI), American Society of Anesthesiologists (ASA) physical status classification, comorbidities (diabetes mellitus [DM], hypertension [HTN], dyslipidemia [DL], chronic kidney disease [CKD], chronic obstructive pulmonary disease [COPD], liver cirrhosis [LC], smoking status, neuropsychiatric medication use, malignancy history, heart disease), tumor maximal diameter, Knosp grade invasiveness, and recurrence status.

After 1:1 propensity score matching, residual imbalances (SMD > 0.25) remained for propensity score (SMD = 0.56), absence of heart disease (SMD = 0.32), and diabetes mellitus (SMD = 0.30), reflecting the intrinsic association between these age-related comorbidities and chronological age that cannot be fully balanced while preserving sample size.
