# Supplementary material for: Surgical outcomes of endoscopic endonasal surgery for nonfunctioning pituitary adenoma in elderly patients: a comprehensive analysis beyond age: Surgery for pituitary adenoma among elderly patients
Source: BMC Endocr Disord. 2026 Feb 12;26:69. doi: 10.1186/s12902-026-02173-6 (PMC12922220; doi:10.1186/s12902-026-02173-6)
Supplement: Supplementary file 2 — Additional file 2: (Figure) Comorbidity distribution by age group: heat map visualization. [file 12902_2026_2173_MOESM2_ESM.pdf]

Additional files 2. Comorbidity distribution by age group: heat map visualization.

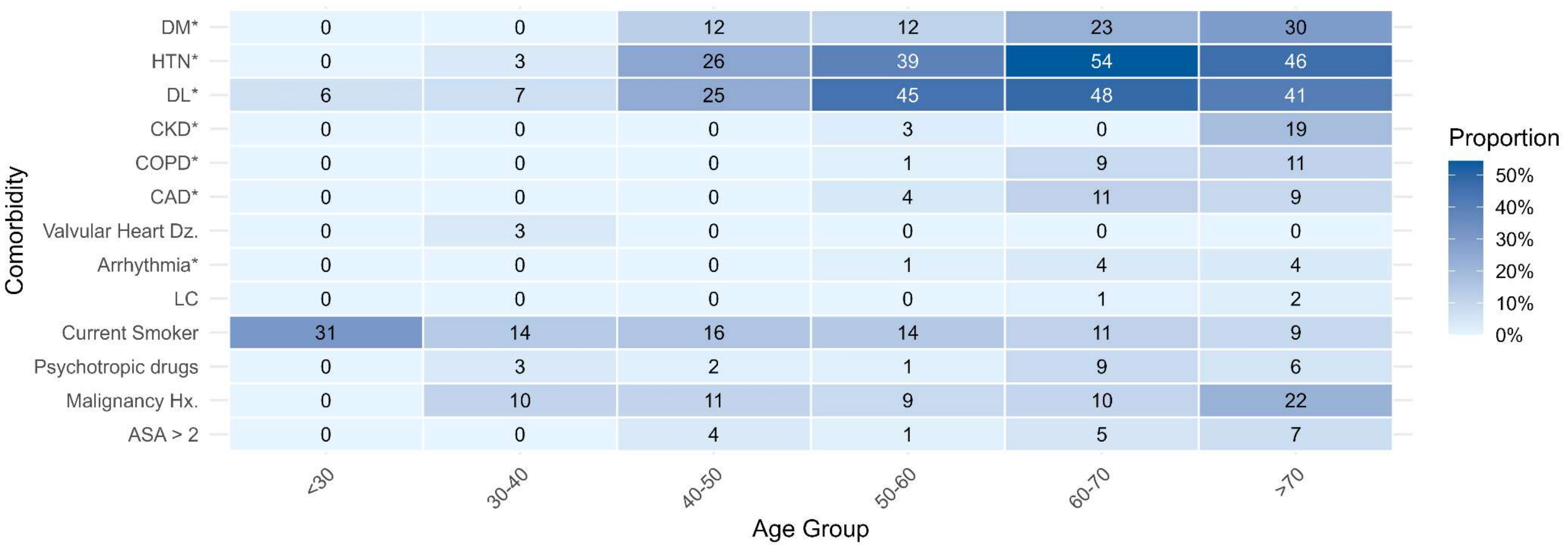

Heatmap showing the proportions of various comorbidities across 10-year age groups (<30, 30-40, 40-50, 50-60, 60-70, >70 years). The color intensity represents the percentage of patients with each comorbidity within each age group, ranging from 0% (light blue) to 50% (dark blue). Asterisks (*) indicate comorbidities with statistically significant age-related trends ($p<0.05$, chi-square test for trend). "ASA > 2" indicates American Society of Anesthesiologists physical status classification class 3 or higher. "Psychotropic drugs" refers to neuropsychiatric medication use.

Abbreviations: HTN, hypertension; DM, diabetes mellitus; DL, dyslipidemia; COPD, chronic obstructive pulmonary disease; CKD, chronic kidney disease; CAD, coronary artery disease; ASA, American Society of Anesthesiologists physical status classification; LC, liver cirrhosis; Hx, history.
