# Supplementary material for: Surgical outcomes of endoscopic endonasal surgery for nonfunctioning pituitary adenoma in elderly patients: a comprehensive analysis beyond age: Surgery for pituitary adenoma among elderly patients
Source: BMC Endocr Disord. 2026 Feb 12;26:69. doi: 10.1186/s12902-026-02173-6 (PMC12922220; doi:10.1186/s12902-026-02173-6)
Supplement: Supplementary file 3 — Additional file 3: (Table) Comorbidities by 10-year age groups. [file 12902_2026_2173_MOESM3_ESM.pdf]

Additional file 3. Comorbidities by 10-year age groups.

	<30 (n=16)	30-40 (n=29)	40-50 (n=57)	50-60 (n=70)	60-70 (n=79)	>70 (n=54)	p[1]
**BMI (kg/m^2)**	23.00 [20.75, 25.50]	25.00 [23.00, 26.75]	25.00 [23.00, 28.00]	26.00 [23.00, 29.00]	26.00 [23.00, 28.00]	26.00 [24.00, 27.00]	0.298[2]
**DM**	0	16 (29.6)	0	7 (12.3)	8 (11.4)	18 (22.8)	**0.001**
**HTN**	0	25 (46.3)	1 (3.4)	15 (26.3)	27 (38.6)	43 (54.4)	**<0.001**
**DL**	1 (6.2)	22 (40.7)	2 (6.9)	14 (24.6)	32 (45.7)	38 (48.1)	**<0.001**
**CKD**	0	10 (18.5)	0	0	2 (2.9)	0	**<0.001[2]**
**COPD**	0	6 (11.1)	0	0	1 (1.4)	7 (8.9)	**0.011[2]**
**LC**	0	1 (1.9)	0	0	0	1 (1.3)	0.784[2]
**Heart disease**	0	7 (13.0)	2 (6.9)	0	4 (5.7)	12 (15.2)	**0.016**
**CAD**	0	5 (9.3)	0	0	3 (4.3)	9 (11.4)	
**Valvular disease**	0	0	1 (3.4)	0	0	0	
**Arrythmia**	0	2 (3.7)	0	0	1 (1.4)	3 (3.8)	
**LC**	0	0	1 (3.4)	0	0	0	0.784
**Smoking**							0.308[2]
**current**	5 (31.2)	5 (9.3)	4 (13.8)	9 (15.8)	10 (14.3)	9 (11.4)	
**Ex-smoker**	2 (12.5)	4 (7.4)	1 (3.4)	5 (8.8)	1 (1.4)	4 (5.1)	
**NP**	0	3 (5.6)	1 (3.4)	1 (1.8)	1 (1.4)	7 (8.9)	0.271[2]
**Malignancy Hx.**	0	12 (22.2)	3 (10.3)	6 (10.5)	6 (8.6)	8 (10.1)	0.108
**ASA**							
**Class 1**	7 (43.8)	0	6 (20.7)	4 (7.0)	4 (5.7)	3 (3.8)	
**Class 2**	9 (56.2)	50 (92.6)	23 (79.3)	51 (89.5)	65 (92.9)	72 (91.1)	
**Class 3**	0	4 (7.4)	0	2 (3.5)	1 (1.4)	4 (5.1)	

Data are presented as n (%)

1 Statistical comparisons were performed using Chi-squared test for categorical variables

2 Kruskal-Wallis test for non-normally distributed continuous variables (marked as "nonnormal"),

3 Fisher's exact test where appropriate

Significant differences ($p < 0.05$) were observed for BMI, diabetes mellitus, hypertension, dyslipidemia, chronic kidney disease, lung disease, and heart disease, demonstrating increasing comorbidity burden with advancing age.

Abbreviations: ASA, American Society of Anesthesiologists; BMI, body mass index; CAD, coronary artery disease; CKD, chronic kidney disease; COPD, chronic obstructive pulmonary disease; DL, dyslipidemia; DM, diabetes mellitus; HTN, hypertension; Hx., history; LC, liver cirrhosis; NP, neuropsychiatric drug.
