# Supplementary material for: Surgical outcomes of endoscopic endonasal surgery for nonfunctioning pituitary adenoma in elderly patients: a comprehensive analysis beyond age: Surgery for pituitary adenoma among elderly patients
Source: BMC Endocr Disord. 2026 Feb 12;26:69. doi: 10.1186/s12902-026-02173-6 (PMC12922220; doi:10.1186/s12902-026-02173-6)
Supplement: Supplementary file 4 — Additional file 4: (Figure) Tumor pathology distribution by lineage and age group: pie charts and stacked bar graphs. [file 12902_2026_2173_MOESM4_ESM.pdf]

Additional file 4. Tumor pathology distribution by lineage and age group: pie charts and stacked bar graphs.

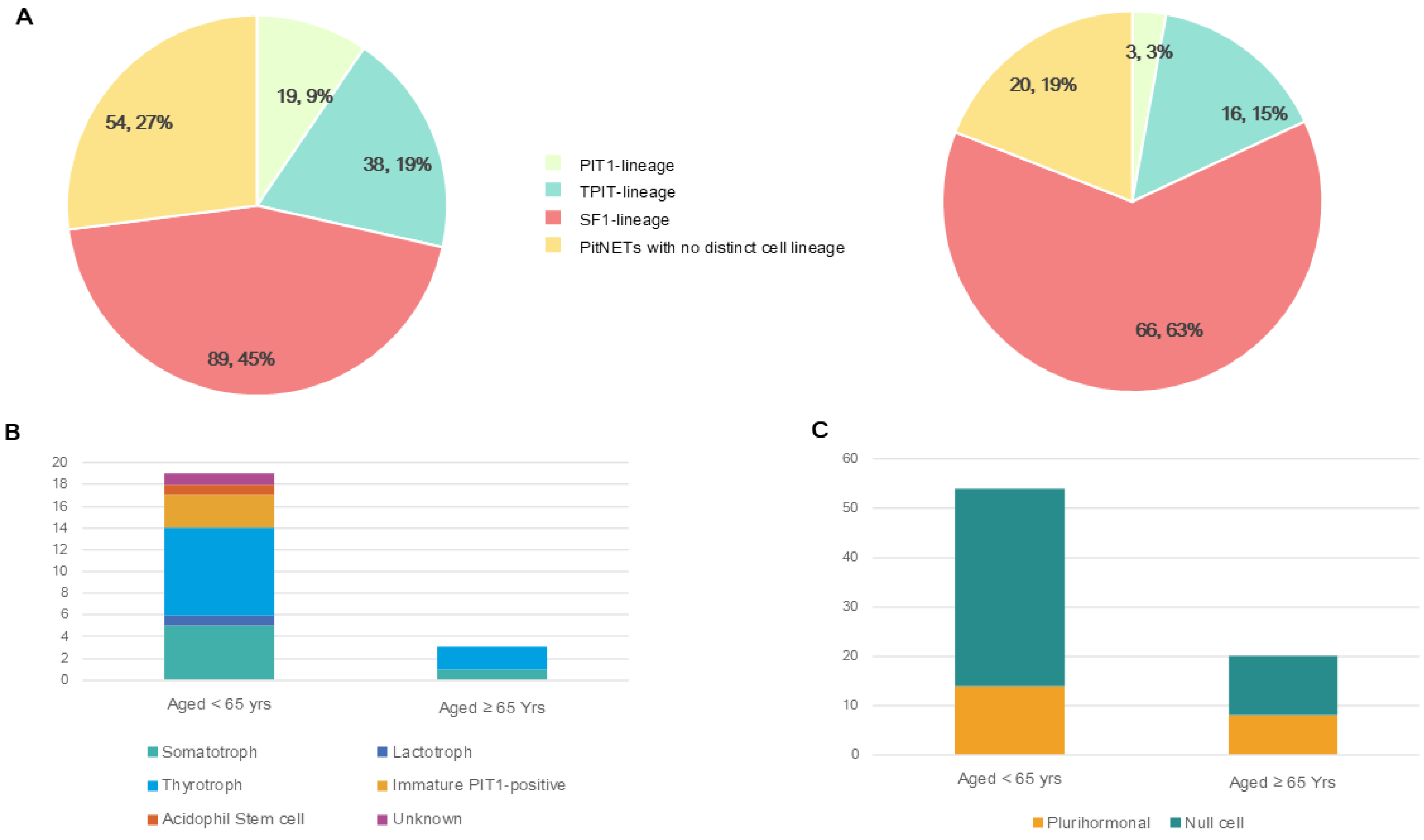

(A) Pie charts showing the overall distribution of pituitary neuroendocrine tumor lineages by age group. Left panel: patients aged <65 years (n=200); right panel: patients aged ≥65 years (n=105). The colors represent different tumor lineages: the PIT1 lineage (light green), the TPIT lineage (teal), the SF1 lineage (red), and PitNETs with no distinct cell lineage (yellow). Elderly patients had a significantly greater proportion of SF1-lineage tumors (gonadotrophs) than younger patients did (62.9% vs. 44.5%, $p=0.011$).

(B) Detailed breakdown of PIT1-lineage tumors by age group, showing specific tumor subtypes. The stacked bar chart displays the distributions of somatotroph (dark blue), lactotroph (orange), thyrotroph (green), immature PIT1-positive (light blue), acidophil stem cell (purple), and unknown PIT1-lineage subtype (light green) tumors. PIT1-lineage tumors were more prevalent in younger patients (9.5% vs. 2.9% in elderly patients).

(C) Detailed breakdown of PitNETs with no distinct cell lineage by age group. The stacked bar chart shows the distribution of plurihormonal (dark blue) and null cell (orange) tumor subtypes. The proportion of these tumors without distinct lineages was greater in younger patients (27% vs. 19% in elderly patients).

Abbreviations: PIT1, POU class 1 homeobox 1 (transcription factor for somatotrophs, lactotrophs, thyrotrophs); PitNET, pituitary neuroendocrine tumor; SF1, steroidogenic factor 1 (transcription factor for gonadotrophs); TPIT, T-box pituitary transcription factor (transcription factor for corticotrophs).
