# Supplementary material for: Surgical outcomes of endoscopic endonasal surgery for nonfunctioning pituitary adenoma in elderly patients: a comprehensive analysis beyond age: Surgery for pituitary adenoma among elderly patients
Source: BMC Endocr Disord. 2026 Feb 12;26:69. doi: 10.1186/s12902-026-02173-6 (PMC12922220; doi:10.1186/s12902-026-02173-6)
Supplement: Supplementary file 5 — Additional file 5: (Table) Detailed tumor pathology distribution and Ki-67 proliferation index by age group. [file 12902_2026_2173_MOESM5_ESM.pdf]

Additional file 5. Detailed tumor pathology distribution and Ki-67 proliferation index by age group.

**Pathology Subtype**	**Overall (n=305)**	**Aged <65 yrs (n=200)**	**Aged ≥65 yrs (n=105)**	**P-value**
**Lineage Distribution**				**0.011**[1]
**PIT1-lineage**	**22 (7.2)**	**19 (9.5)**	**3 (2.9)**	**0.038**[2]
Somatotroph	6 (2.0)	5 (2.5)	1 (1.0)	0.667
Lactotroph	1 (0.3)	1 (0.5)	0 (0.0)	1.000
Thyrotroph	10 (3.3)	8 (4.0)	2 (1.9)	0.536
Immature PIT1-positive	3 (1.0)	3 (1.5)	0 (0.0)	0.551
Acidophil Stem cell	1 (0.3)	1 (0.5)	0 (0.0)	
Unknown	1 (0.3)	1 (0.5)	0 (0.0)	
**TPIT-lineage**	**54 (17.7)**	**38 (19.0)**	**16 (15.2)**	0.484
Corticotroph		38 (19.1)	16 (15.2)	
**SF1-lineage**	**155 (50.8)**	**89 (44.5)**	**66 (62.9)**	**0.003**
Gonadotroph		89 (44.7)	66 (62.9)	
**PitNETs with no distinct cell lineage**	**74 (24.3)**	**54 (27.0)**	**20 (19.0)**	**0.133**
Plurihormonal	22 (7.2)	14 (7.0)	8 (7.6)	0.803
Null cell	52 (17.0)	40 (20.1)	12 (11.4)	0.063
**Ki-67**	2.2 [1.4,3.4]	2.4 [1.6, 3.8]	1.8 [1.0, 2.6]	**<0.001**[3]

Data are presented as n (%) for categorical variables and median [interquartile range] for continuous variables (Ki-67 proliferation index).
[1]P-values from chi-square test comparing categorical distributions between age groups.
[2]P-values for individual rows were calculated comparing "Specific Subtype vs. Others" between age groups using Fisher's exact test.
[3]P-value from Mann-Whitney U test comparing Ki-67 distributions between age groups.

Tumor lineage classification based on immunohistochemistry for pituitary transcription factors: PIT1 (POU class 1 homeobox 1) for somatotrophs, lactotrophs, and thyrotrophs; TPIT (T-box pituitary transcription factor) for corticotrophs; SF1 (steroidogenic factor 1) for gonadotrophs. Tumors negative for all lineage markers were classified as plurihormonal (positive for multiple hormones without specific transcription factor) or null cell (negative for hormones and transcription factors). Significant differences were observed in the distribution of PIT1-lineage tumors (p=0.011) and SF1-lineage tumors (p=0.003), with elderly patients showing a higher proportion of gonadotroph adenomas and younger patients showing more PIT1-lineage tumors. Ki-67

proliferation index was significantly higher in younger patients (median 2.40% vs. 1.80%, $p<0.001$).

Abbreviations: PIT1, POU class 1 homeobox 1; PitNET, pituitary neuroendocrine tumor; SF1, steroidogenic factor 1; TPIT, T-box pituitary transcription factor.
