# Supplementary material for: Surgical outcomes of endoscopic endonasal surgery for nonfunctioning pituitary adenoma in elderly patients: a comprehensive analysis beyond age: Surgery for pituitary adenoma among elderly patients
Source: BMC Endocr Disord. 2026 Feb 12;26:69. doi: 10.1186/s12902-026-02173-6 (PMC12922220; doi:10.1186/s12902-026-02173-6)
Supplement: Supplementary file 6 — Additional file 6: (Table) Surgical outcomes and complications by 10-year age groups. [file 12902_2026_2173_MOESM6_ESM.pdf]

Additional file 6. Surgical outcomes and complications by 10-year age groups.

	Overall (N=305)	<30 (n=16)	30-40 (n=29)	40-50 (n=57)	50-60 (n=70)	60-70 (n=79)	>70 (n=54)	p
**GTR**	259 (84.9)	14 (87.5)	24 (82.8)	52 (91.2)	58 (82.9)	65 (82.3)	46 (85.2)	0.757[1]
**Any Complications**	175 (57.4)	6 (37.5)	15 (51.7)	31 (54.4)	42 (60.0)	48 (60.8)	33 (61.1)	0.543[1]
**Readmission**	13 (4.3)	1 (6.2)	0	3 (5.3)	3 (4.3)	3 (3.8)	3 (5.6)	0.870[3]
**Reoperation**	6 (2.0)	0	0	1 (1.8)	3 (4.3)	2 (2.5)	0	0.7322[2]
**LOS (days)**	4	4	4	4	4	4	4	0.396[3]
**ICU (days)**	1	1	1	1	1	1	1	0.588[3]
**Prolonged stay**	71 (23.3)	4 (25.0)	3 (10.3)	11 (19.3)	17 (24.3)	24 (30.4)	12 (22.2)	0.352[1]
**Hyponatremia**	81 (26.6)	4 (25.0)	7 (24.1)	12 (21.1)	18 (25.7)	21 (26.6)	19 (35.2)	0.688[1]
**DI**	96 (31.5)							0.959[1]
Transient	75 (24.6)	2 (12.5)	7 (24.1)	14 (24.6)	18 (25.7)	20 (25.3)	14 (25.9)	
Persistent	21 (6.9)	1 (6.2)	1 (3.4)	4 (7.0)	6 (8.6)	7 (8.9)	2 (3.7)	
**Delirium**	14 (4.6)	0	0	3 (5.3)	4 (5.7)	3 (3.8)	4 (7.4)	0.740[2]
**Pneumonia**	1 (0.3)	0	0	0	0	0	1 (1.9)	0.325[2]
**DVT**	0 (0.0)	0	0	0	0	0	0	
**SSI**	9 (3.0)	0	0	1 (1.8)	1 (1.4)	7 (8.9)	0	0.057[2]

Data are presented as n (%) for categorical variables and median [interquartile range] for continuous variables.

Outcome definitions: GTR, gross total resection; Hospital length of stay (LOS), total hospital length of stay; ICU, intensive care unit length of stay; DI, diabetes insipidus; DVT, deep vein thrombosis; SSI, surgical site infection. Prolonged stay was defined as hospital length of stay ≥5 days. Hyponatremia was defined as serum sodium <135 mEq/L within 3 months postoperatively. Transient DI was defined as requiring treatment but resolving within 3 months, while persistent DI continued beyond 3 months. Hospital length of stay and intensive care unit stay showed uniform distributions across all age groups (median 4 days and 1 day, respectively, with IQR of 4-4 and 1-1 for all groups).

[1]Statistical comparisons were performed using Chi-squared test for categorical variables, No statistically significant differences were observed between age groups for any outcome measure (all p > 0.05).

[2]Kruskal-Wallis test for non-normally distributed continuous variables

[3]Fisher's exact test where appropriate (used when expected cell frequencies <5).
