# Supplementary material for: Surgical outcomes of endoscopic endonasal surgery for nonfunctioning pituitary adenoma in elderly patients: a comprehensive analysis beyond age: Surgery for pituitary adenoma among elderly patients
Source: BMC Endocr Disord. 2026 Feb 12;26:69. doi: 10.1186/s12902-026-02173-6 (PMC12922220; doi:10.1186/s12902-026-02173-6)
Supplement: Supplementary file 7 — Additional file 7: (Table) Multivariable regression models for postoperative outcomes. [file 12902_2026_2173_MOESM7_ESM.pdf]

Additional file 7. Multivariable regression models for postoperative outcomes.

Variables	GTR[1]	Overall Complication[1]	Surgical Site Infection[1]	Hospital Stay[2]	Hyponatremia[1]
**Patient Factors**					
Age	0.96 (0.64-1.44)	1.25 (0.95-1.65)	1.35 (0.44-4.72)	1.00 (0.94-1.06)	1.06 (0.80-1.41)
Sex (Female)	**0.48 (0.24-0.96) ***	1.45 (0.90-2.34)	-	-	-
BMI	-	-	**1.24 (1.02-1.53) ***	**1.02 (1.01-1.04) ****	**0.92 (0.84-0.99) ***
**Comorbidities**					
DM	-	-	-	-	-
COPD	-	-	-	**1.34 (1.06-1.70)***	-
CAD	-	-	**12.35 (1.88-104.58) ****	1.14 (0.98-1.32)	2.58 (0.95-7.00)
Arrythmia	-	-	**29.31 (1.80-473.41) ***	1.05 (0.85-1.30)	-
DL	-	**0.58 (0.34-0.97) ***	-	-	-
ASA Gr (>2 vs ≤2)	-	1.97 (0.81-4.78)	-	-	-
**Tumor Characteristics**					
Recurred Tumor	**0.22 (0.10-0.49) ***	-	4.53 (0.77-29.08)	**1.20 (1.04-1.38) ***	-
Knosp Gr 4 (vs 0-3)	**0.06 (0.01-0.48) ***	-	-	-	-
**Surgical Factors**					
IntraOp CSF	-	-	**5.90 (1.28-38.16) ***	**1.20 (1.06-1.36) ****	1.65 (0.95-2.85)
Transient DI	-	-	-	-	**2.00 (1.06-3.64) ***
Persistent DI					**3.87 (1.20-12.49) ***
Any Complication	-	-	-	**1.38 (1.23-1.54)*****	-
Approach (vs combined)[3]	-	-	-	**(Significant) ***	-
Trans-sellar only				**0.41 (95% CI: 0.22-0.76)*****	
Sagittal Extension				**0.41 (95% CI: 0.22-0.76)****	
Coronal Extension				**0.35 (95% CI: 0.18-0.66)****	

Values are presented as odds ratios (OR) or rate ratios (RR) with 95% confidence intervals (CI). Each column represents a separate multivariable regression model tailored to the specific outcome. Variables shown with "—" were not included in that particular model based on clinical relevance and preliminary univariable analysis ($p>0.10$ threshold for inclusion). Age was not a significant independent predictor of any surgical outcome after adjusting for comorbidities, tumor characteristics, and surgical factors. Instead, specific modifiable factors (dyslipidemia, surgical approach, intraoperative CSF leak) and tumor-related factors (Knosp grade, recurrence) emerged as key determinants of outcomes. *$p<0.05$, **$p<0.01$, ***$p<0.001$

[1] Odds ratios derived from logistic regression (Firth's penalized likelihood method for surgical site infection due to rare events; standard maximum likelihood for other binary outcomes).

[2] Rate ratio derived from negative binomial regression for hospital length of stay (count outcome with overdispersion).

[3] Surgical approach significantly predicted hospital stay (overall Wald test $p<0.001$). Reference category: combined multicorridor approach. Single-corridor approaches (trans-sellar, sagittal extension, coronal extension) were each associated with significantly shorter hospital stays.

Abbreviations: ASA Gr, American Society of Anesthesiologists physical status grade; BMI, body mass index; CAD, coronary artery disease; CI, confidence interval; COPD, chronic obstructive pulmonary disease; CSF, cerebrospinal fluid; DI, diabetes insipidus; DL, dyslipidemia; DM, diabetes mellitus; GTR, gross total resection; IntraOp, intraoperative; OR, odds ratio; RR, rate ratio.
