# Supplementary material for: Surgical outcomes of endoscopic endonasal surgery for nonfunctioning pituitary adenoma in elderly patients: a comprehensive analysis beyond age: Surgery for pituitary adenoma among elderly patients
Source: BMC Endocr Disord. 2026 Feb 12;26:69. doi: 10.1186/s12902-026-02173-6 (PMC12922220; doi:10.1186/s12902-026-02173-6)
Supplement: Supplementary file 8 — Additional file 8: (Table) Pairwise comparisons of endocrinological status changes by 10-year age groups. [file 12902_2026_2173_MOESM8_ESM.pdf]

Additional file 8. Pairwise comparisons of endocrinological status changes by 10-year age groups.

Comparison	Corticotroph	Lactotroph	Somatotroph	Gonadotroph	Thyrotroph
>70 vs 60-70	1.000	1.000	1.000	1.000	1.000
>70 vs 30-40	1.000	0.380	**0.039**	**0.003**	1.000
>70 vs 40-50	1.000	0.152	0.810	**0.038**	1.000
>70 vs 50-60	1.000	1.000	0.369	0.204	1.000
>70 vs <30	1.000	1.000	0.558	**0.002**	1.000
60-70 vs 30-40	**0.005**	0.415	0.207	**0.011**	1.000
60-70 vs 40-50	1.000	0.291	1.000	**0.047**	1.000
60-70 vs 50-60	0.137	1.000	1.000	0.870	1.000
60-70 vs <30	1.000	1.000	0.536	0.007	1.000
30-40 vs 40-50	0.705	1.000	1.000	1.000	1.000
30-40 vs 50-60	0.412	1.000	1.000	1.000	1.000
30-40 vs <30	1.000	1.000	1.000	1.000	1.000
40-50 vs 50-60	1.000	1.000	1.000	1.000	1.000
40-50 vs <30	1.000	1.000	1.000	1.000	1.000
50-60 vs <30	1.000	1.000	1.000	1.000	1.000

Adjusted p-values from pairwise Fisher's exact tests comparing endocrinological status changes between age groups (Holm-Bonferroni correction for multiple comparisons).

Status changes: improved (deficit→normal), worsened (normal→deficit), persistent deficit, or maintained normal.

Bold values (p<0.05) indicate significant differences. Gonadotroph and somatotroph axes showed age-related recovery patterns, with elderly patients (>70, 60-70 years) demonstrating reduced improvement compared to younger groups. Lactotroph, and thyrotroph axes showed age-independent recovery.
