# Supplementary material for: Surgical outcomes of endoscopic endonasal surgery for nonfunctioning pituitary adenoma in elderly patients: a comprehensive analysis beyond age: Surgery for pituitary adenoma among elderly patients
Source: BMC Endocr Disord. 2026 Feb 12;26:69. doi: 10.1186/s12902-026-02173-6 (PMC12922220; doi:10.1186/s12902-026-02173-6)
Supplement: Supplementary file 9 — Additional file 9: (Table) Age thresholds for hormone recovery by axis: maximally selected rank statistics and bootstrap validation. [file 12902_2026_2173_MOESM9_ESM.pdf]

Additional file 9. Age thresholds for hormone recovery by axis: maximally selected rank statistics and bootstrap validation.

Hormone Axis	Maxstat Optimal Cut-off	Maxstat P-value	Bootstrap Mean Cutoff (95% CI)[1]
**Composite Outcome[2]**	57	< 0.001	60.1 (42 - 71)
**Individual Axes**			
**Gonadotroph**	46	< 0.001	46.5 (37 - 60)
**Somatotroph**	55	0.007	56.3 (42 - 69)
**Corticotroph[3]**	64[3]	0.988	59.2 (46- 74)
**Lactotroph[4]**	36[3]	0.056	48.7 (31 - 70)

[1] Bootstrap validation was performed with 1,000 replications. "Mean Peak" represents the average optimal cut-off age identified across bootstrap samples.

[2]Composite Recovery refers to the recovery of any deficient hormone axis postoperatively.

[3] Corticotroph axis: Maxstat identified a cutoff at age 64, but P-value was not significant (0.988), indicating no statistically reliable age threshold for corticotroph recovery.

[4]Lactotroph axis: Maxstat identified a cutoff at age 36 with borderline significance (P=0.056), suggesting a possible but statistically weak age effect.

Thyrotroph axis analysis is not shown. No significant age threshold was identified (Maxstat P=0.758), and bootstrap validation was not performed due to insufficient recovery events (n<5).
