# Supplementary material for: Surgical outcomes of endoscopic endonasal surgery for nonfunctioning pituitary adenoma in elderly patients: a comprehensive analysis beyond age: Surgery for pituitary adenoma among elderly patients
Source: BMC Endocr Disord. 2026 Feb 12;26:69. doi: 10.1186/s12902-026-02173-6 (PMC12922220; doi:10.1186/s12902-026-02173-6)
Supplement: Supplementary file 10 — Additional file 10: (Table) Predictors of hormone recovery for each pituitary axis: multivariable logistic regression models. [file 12902_2026_2173_MOESM10_ESM.pdf]

Additional file 10. Predictors of hormone recovery for each pituitary axis: multivariable logistic regression models.

**Variable**	**Adjusted OR (95% CI)**	**P-value**
**Gonadtroph Axis**		
**Age[1]**	**0.93 (0.89 – 0.96)**	**< 0.001**
Female Sex	2.31 (0.97 – 5.55)	0.058
**BMI[1]**	**0.86 (0.76 – 0.97)**	**0.013**
Recurrent Tumor	0.13 (0.01 – 0.74)	0.060
Gross Total Resection[2]	1.70 (0.40 – 8.70)	0.493
Intraop CSF Leak	0.82 (0.33 – 1.97)	0.666
Pathology[3]		
Corticotroph	0.58 (0.17 – 1.96)	0.391
**Null cell**	**0.27 (0.08 – 0.82)**	**0.029**
**Plurihormonal**	**0.08 (0.00 – 0.51)**	**0.025**
**Thyrotroph**	**0.07 (0.00 – 0.67)**	**0.037**
Others[4]	0.21 (0.02 – 1.42)	0.137
**Somatotroph Axis**		
**Age[1]**	**0.97 (0.94 – 0.99)**	**0.022**
**Female Sex**	**2.79 (1.24 – 6.41)**	**0.014**
Recurrent Tumor	0.25 (0.04 – 1.06)	0.098
Gross Total Resection[2]	2.03 (0.52 – 10.49)	0.340
Intraoperative CSF Leak	2.31 (0.99 – 5.38)	0.050
**Corticotroph Axis**		
Age[1]	1.01 (0.95 – 1.08)	0.713
**Female Sex**	**5.24 (1.11 – 30.73)**	**0.045**
**Ki-67 Index[1]**	**1.47 (1.10 – 2.08)**	**0.015**
Pathology[1]		
Corticotroph	1.57 (0.16 – 12.69)	0.672
Null cell	2.27 (0.41 – 12.09)	0.332
Plurihormonal	16.13 (1.89 – 171.25)	0.013
**Lactotroph Axis**		
Age	1.01 (0.93 – 1.10)	0.820
Tumor Volume[1]	0.99 (0.97 – 1.00)	0.102
Gross Total Resection[2]	1.84 (0.18 – 19.50)	0.598
Pathology[3]		
Corticotroph	0.33 (0.01 – 3.47)	0.385
Null cell	0.32 (0.01 – 4.45)	0.407
Others[4]	0.09 (0.00 – 3.43)	0.169

[1]Continuous variables (Age, BMI, Tumor Volume, Ki-67) were standardized (Z-score transformed); Adjusted Odds Ratios (aOR) represent the effect per 1-standard deviation increase.
[2]Reference category for Resection was non-GTR. *P*-values < 0.05 are indicated in bold.
[3]Reference category for Pathology was Gonadotroph subtype.
[4]Others includes somatotroph, lactotroph, and unclassified PIT1-lineage tumors (combined due to small sample sizes: n<10 each).

Age remained a significant independent predictor only for gonadotroph (OR 0.93 per SD, p<0.001) and somatotroph (OR 0.97 per SD, p=0.022) axes, confirming age-related decline in these axes is intrinsic to aging rather than confounded by tumor characteristics or surgical factors. Female sex showed protective effects for somatotroph (OR 2.79, p=0.014) and corticotroph (OR 5.24, p=0.045) recovery, likely reflecting sex hormone interactions and higher baseline growth hormone levels in

women. Corticotroph, lactotroph, and thyrotroph axes demonstrated age-independent recovery patterns.
Thyrotroph axis: not shown due to insufficient recovery events (n<5) precluding reliable multivariable modeling.

Model performance: Gonadotroph (AUC 0.821, Pseudo R^2 0.265), Somatotroph (AUC 0.722, Pseudo R^2 0.124), Corticotroph (AUC 0.811, Pseudo R^2 0.212), Lactotroph (AUC 0.777, Pseudo R^2 0.205).

Abbreviations: OR, odds ratio; CI, confidence interval; BMI, body mass index; CSF, cerebrospinal fluid; AUC, area under the curve; ref, reference category.
